# Improving the Physical Health of Psychiatric Hospital Residents: An Evaluation of an Obesity Education Program for Mental Health Professionals

**DOI:** 10.3390/healthcare10101851

**Published:** 2022-09-23

**Authors:** Ellis E. Opusunju, Patrick A. Palmieri, Karen A. Dominguez-Cancino, Sebastian Jaque-Ortiz, Diane K. Whitehead

**Affiliations:** 1 Twin Valley Behavioral Healthcare, 2200 W. Broad St., Columbus, OH 43223, USA; 2 College of Nursing, Walden University, 100 S. Washington Ave., Suite 1210, Minneapolis, MN 55401, USA; 3 South American Center for Qualitative Research, Universidad Norbert Wiener, Av. Arequipa 444, Lima 15046, Peru; 4 College of Graduate Health Studies, A.T. Still University, 800 West Jefferson Street, Kirksville, MO 63501, USA; 5 EBHC South America: A JBI Affiliated Group, Calle Cartavio 406, Lima 15023, Peru; 6 Center for Global Nursing, Texas Woman’s University, 6700 Fannin St., Houston, TX 77030, USA; 7 Addiction Study Program, Université de Sherbrooke, 150, Place Charles-Le Moyne, Bureau 200, Longueuil, QC J4K 0A8, Canada; 8 Escuela de Salud Pública, Universidad de Chile, Av. Independencia 939, Santiago 8380888, Chile; 9 Hospital Dr. Exequiel González Cortés, Gran Av. José Miguel Carrera 3300, Santiago 8900295, Chile

**Keywords:** obesity, overweight, mental health, psychiatric hospital, mental health professionals, professional education, PARiHS framework, advising and treating overweight and obese patient questionnaire, implementation science, evidence-based practice

## Abstract

Background: People living with mental health disorders are at increased risk for developing obesity due to poor diet, physical inactivity, and antipsychotic medications. In the United States, the obesity rate is 36% in the general population and more than 50% for people living with mental health disorders. Although mental health clinicians concentrate on managing psychiatric disorders, they seldom recognize the gradual increase in body mass index of their patients. The result is a disconnection between the clinical management of psychiatric disorders and the medical management of obesity. Purpose: This study assessed the effectiveness of an evidence-based education program for improving the obesity management practices of mental health clinicians caring for residents at a state psychiatric hospital. Methods: This was a quasi-experimental study design with a pretest and posttest evaluation. Convenience sampling was used to recruit mental health professionals, or clinicians, at a large psychiatric hospital in the Southern region of the United States. Data was collected with the Advising and Treating Overweight and Obese Patient questionnaire (17 items). Data analysis included descriptive and inferential statistics. The findings were reported in accordance with the TREND and GREET guidelines. Results: The education program was completed by 50 MHCs. The pretest indicated that 76% of MHCs were not involved in helping obese residents manage their weight, but the posttest indicated 90% were involved. There was a significant increase in MHC knowledge about obesity management and reported actions 90-days after the program. MHCs were unable to arrange follow-up visits for residents, a task not directly within their control. Conclusions: Mental health clinicians reported increased knowledge and improved clinical practice after an education program. Because the outcomes were reported at 90-days after the program, further research needs to evaluate the longitudinal impact of this type of program, where the reported behaviors are correlated to process and clinical outcome measures for obesity.

## 1. Introduction

As the second leading cause of preventable death in the United States [1], obesity is a national public health crisis [2]. An obese person, defined by a body mass index (BMI) of 30 or greater [3,4], will have, on average, a shorter life expectancy of 14 years [5]. People living with a mental disorder are twice as likely to be obese than the general population [6]. With a higher prevalence of morbidity [7] and a two to three times higher mortality rate than the general population [8,9], obesity is a leading health problem for people living with a mental disorder [10]. The overall impact can be illustrated through the age-standardized, disability-adjusted life years of 2235 years (depressive disorders), 551 years (anxiety disorders), and 288 years (schizophrenia) per 100,000 population in the United States [11].

Approximately 1 in 25 people is diagnosed with a serious mental disorder [12], including 8 million Americans with bipolar disorder or schizophrenia [13]. This population experiences poor nutrition, sedentary lifestyle, substance abuse, overeating, excessive tobacco use, and poor coordination of care that contributes to an increased risk for obesity [14,15,16,17,18]. In addition, the population requires antipsychotic medications that potentiate weight gain [19]. Capodaglio et al. [20] reported that 42% of people with schizophrenia had a BMI greater than 27 when compared to 27% of the general population. Because of lifestyle decisions resulting in weight gain in this population [21], obesity causes a reduced life expectancy of 7 to 20 years [22,23,24] and doubles the risk for mortality [25,26]. Obesity for people living with mental disorders is a complex issue requiring evidence-based prevention strategies [27] implemented by multidisciplinary teams [28].

### 1.1. Psychiatric Hospitals

The weight gain of residents in psychiatric hospitals has been discussed for decades [29] as a concern for chronic diseases [30], yet little has improved for residents in this obesogenic [31] or obesity promoting environment [32]. The residents are often inactive or sedentary [33] with diets high in fat and carbohydrates that contribute to metabolic and nutritional disorders [21]. According to McKibbin et al. [34], the problem is made more complicated by the range of mental disorders, such as bipolar disorder, depression, schizophrenia, and anxiety, that increase the risk for obesity in different ways. Yet, there continues to be a practice gap in psychiatric hospitals because clinicians are not identifying residents at risk for obesity and implementing timely medical management strategies [19].

The risks associated with obesity at psychiatric hospitals may result from five causes addressed in other settings. First, residents enter the hospital with higher rates of chronic conditions than the general population [35]. Second, residents have poor dietary habits [34]. Third, residents are often unable to synthesize health-related information, receive inadequate dietary education, and lack familiarity with healthy eating habits [36]. Fourth, the profile of prescribed psychiatric medications, especially the older and less expensive medications, often have adverse side effects that cause weight gain [14]. Fifth, residents have low expectations about an appropriate BMI because of alterations in body image and inadequate support from friends and family members [37,38]; therefore, they make no attempt to lose weight. Despite frequent interactions with the health system, these five factors directly contribute to people living with mental disorders becoming obese.

### 1.2. Conceptual Framework

The Promoting Action on Research Implementation in Health Care Services [39], or PARiHS, is a widely used [40] conceptual framework for guiding the development, implementation, and evaluation of projects that advance evidence-based practice [41], including mental health [42]. The PARiHS framework explicates the factors necessary to successfully implement evidence into practice within a complex adaptive environment [43]. The framework has three principal factors: evidence, context, and facilitation [44]. For the framework to be successful [45], there needs to be a clear understanding about the evidence [46] to be applied to a well-defined problem [47]. Moreover, the context for change needs to be assessed [41,43], and the best strategies for effective facilitation [48] need to be applied to successfully implement change [49]. For this study, the PARiHS framework was adopted for a task-oriented approach [50] to guide the implementation and measurement [51] of an evidence-based education program to change the clinical practice of mental health professionals, or clinicians, in a psychiatric hospital. The approach resulted in problem identification, evidence review, education program intervention, readiness assessment, program facilitation, and outcome measurement.

### 1.3. Purpose

The current evidence and best practices about obesity management for residents of psychiatric hospitals suggest the topic has not been widely explored [52], as the published literature is sparse [53]. However, more research has been recommended to investigate the benefits associated with mental health clinicians administering weight management programs [54]. To address the gap in the literature, the perceptions, knowledge, and practices of mental health clinicians about clinically managing obesity requires further exploration [28,53]. As such, the purpose of this study was (1) to understand the perceptions and practices of mental health clinicians working in a psychiatric hospital about managing resident obesity and (2) to evaluate the effectiveness of an evidence-based education program to improve clinician knowledge about identifying overweight residents and implementing clinical management strategies as part of their daily work.

## 2. Methods

### 2.1. Study Design

This pilot study used a quasi-experimental design [55] with an educational program intervention [56] incorporating a pretest and posttest evaluation [57]. The study design was considered appropriate because of limited published evidence about educational programs for obesity management in public psychiatric hospitals. Limitations related to the pretest and posttest evaluation, such as determining causality and establishing generalizability [58,59], were unavoidable because of process considerations and ethical requirements. The study was designed and reported according to Transparent Reporting of Evaluations with Nonrandomized Designs [60], TREND, statement. These 21 criteria are recommended for guiding the design and reporting of evaluation studies [61], including educational interventions [62]. In addition, the educational intervention was reported according to the Reporting Evidence-Based Practice Educational Interventions and Teaching [63], GREET, statement. These 17 criteria are recommended for designing and reporting educational interventions focused on evidence-based practice.

### 2.2. Setting and Sample

The study was conducted at the beginning of 2017 at a large state psychiatric hospital, with approximately 200 beds, located in Southern region of the United States. The hospital is responsible for people living with mental disorders who cannot be cared for in an ambulatory setting because of the severity of their condition. Most residents had been hospitalized for at least six months, and the average length of stay was nine months. The resident population was almost equally men and women, aged between 18 and 65 years, and 55% White, 40% African American, and 5% Hispanic.

A convenience sampling strategy was implemented using email invitations to reach a sample goal of 50 clinicians. The inclusion criteria were licensed mental health clinicians who had prescriptive authority and were credentialed to provide primary care and/or mental health services at the hospital. Clinician profile data was not collected because of restrictions for protecting participant confidentiality.

### 2.3. Data Collection

All participants completed a pretest questionnaire, the one-hour educational program, and a posttest questionnaire in a conference room at the psychiatric hospital. The pretest questionnaire was completed before the educational program. Then, all the participants completed the educational program during the same session. Finally, the posttest questionnaire was completed 90-days after the educational program. Both pretest and posttest questionnaires were returned in sealed unmarked envelopes to a secure box. The participants were instructed to return a blank questionnaire in the envelope at any point during the study if they decided to stop participating. All participants gave written informed consent. No financial incentives were provided for participation.

### 2.4. Continuing Education Program

The continuing education program was developed from a scoping review [53] to identify the best practices and current evidence about obesity management in the context of mental health and psychiatric institutions. The content development and presentation design was guided by recommendations for continuing education programs for health professions [64]. Educational strategies to engage the participants included the repetition of key concepts, clinical examples, group discussion, and focused questions [64,65]. The five learning objectives included the following: (1) Describe the relationship between obesity and mental health disorders; (2) Discuss the role of psychiatric clinicians in the medical management of obesity; (3) Identify the residents at risk for obesity in the facility; (4) Implement the evidence-based recommendations for obesity management within the context of mental health; and (5) Analyze the role conflicts and work limitations impeding the management of obesity in a psychiatric hospital. The content did not include topics such as weight-based stereotypes, discrimination, and explicit and implicit bias [66], because the program was developed before guidelines designed to reduce the stigmatization associated with obesity in clinical management and research. The program recognized the participants are self-directed, experiential, and problem-solving adult learners seeking knowledge that is relevant to their clinical practice [64,67]. The program was presented by a certified nurse practitioner with experience in weight management.

### 2.5. Instrument

The Advising and Treating Overweight and Obese Patient (ATOOP) questionnaire [68], a valid and reliable 17-item instrument [52], was used to assess the perceptions of mental health clinicians about their ability to identify, advise, and manage obese residents (Supplemental File S2). Permission to use the instrument was obtained from the developer. The ATOOP questionnaire has a good reliability coefficient (*r* = 0.81), including for efficacy expectation (*r* = 1.00), outcome expectation (*r* = 0.88), and barriers *(r* = 0.95). Two additional items were added after the ATOOP questionnaire to assess previous obesity management education and training. The complete 19-item instrument was validated, face and content [69,70], by a panel of seven experts for application with mental health clinicians practicing in a psychiatric hospital. Because the instrument was developed before new guidelines about “person first language” for obesity [71,72], an assessment was not completed to identify and revise items that used stigmatizing language.

### 2.6. Data Analysis

The data were entered into an Excel spreadsheet to organize and clean. Analyses were performed in Statistical Analysis System (SAS, version 9.4) software [73]. Descriptive statistics were calculated, including mean difference in responses before and after the educational program with an associated confidence interval of 95% (CI 95%) for numerical variables and a positive or negative change in responses for categorical variables. A positive change corresponded with an improvement in knowledge or practice following the educational program. A detailed analysis was also provided for the core concepts for the study. Paired-sample *t* and McNemar tests [74] were completed to measure pretest and posttest differences between groups [75,76]. An α-level of 0.05 was considered significant.

### 2.7. Ethical Considerations

The project was approved by the psychiatric hospital and the institutional review board at the participating university. Before data collection, all participants were provided with written information about the study. As required by the hospital, no personally identifiable information, including demographic data, was collected during the study. Individual data were aggregated for group reporting. The de-identified data was securely stored on a password-protected computer hard drive with restricted access. There were no breaches in confidentiality and no reported adverse events.

## 3. Results

A total of 50 mental health clinicians responded to the questionnaire before (pretest) and 90-days (posttest) after the educational program. This accounted for 100% of the total responses. There were no incomplete or blank questionnaire returned and no deviations from the study protocol. Although the pretest questionnaire indicated that most clinicians were not involved in helping overweight and obese residents manage their weight, the posttest questionnaire showed that most clinicians became involved in obesity management after the educational program, which was a statistically significant finding. Overall, 15 of the 16 items were significantly improved after the educational program (see Table 1). Additional data analyses, including, bar charts and histograms are provided in Supplemental File S2.

### 3.1. Engagement in Weight Management

The first questionnaire item provided an overall assessment of the clinician’s perspective about assisting overweight and obese patients with losing weight. Before the educational program, 76% of participants most frequently indicated they had not seriously thought about assisting their overweight and obese patients with losing weight. After the educational program, 42% of the participants indicated they had been thinking about assisting their overweight an obese patient with losing weight within the next six months. Importantly, 48% of the participants had developed formal plans or actively began assisting their patients with losing weight. Table 2 provides the item response frequency.

### 3.2. Confidence and Ability to Engage in Weight Management

After the educational program, the participants were more confident in their ability to ask patients if they were concerned about their weight, advise patients about weight management, assess patient willingness to lose weight, assist patients with losing weight, and arrange for follow-up visits for patients; these findings were statistically significant. Although there was a statistically significant improvement in participant confidence in arranging follow-up visits for patients, half the participants were only slightly confident, and very few were confident (14%) or highly confident (2%). Table 3 provides the item response frequency.

**Table 1:** Item Analysis of Obesity Management Knowledge and Practices (*n* = 50).

No.	Items	Pretest and Posttest	*p*-Value
1	Which of the following statements best describes your position toward assisting overweight/obese patients to lose weight?	Positive *	<0.0001
2	What percentage of your visibly overweight/obese patients do you identify and document their weight status?	43.8%(CI95% 37.64–49.95)	<0.0001
3	What percentage of your visibly overweight/obese patients do you give a clear, strong, and personalized message urging them to lose weight?	53.2%(CI 95% 48.15–58.25)	<0.0001
4	What percentage of you overweight/obese patients do you assess whether they are willing to make an effort to lose weight?	52.6%(CI95% 47.98–57.22)	<0.0001
5	What percentage of your overweight/obese patients, who are interested in attempting to lose weight do you or your staff use behavioral counseling to help them lose weight?	48.0%(CI95% 42.52–53.48)	<0.0001
6	What percentage of you overweight/obese patients do you assist by encouraging them to use problem-solving skills for weight loss?	48.0%(CI95% 42.93–53.07)	<0.0001
7	What percentage of your overweight/obese patients do you assist by providing and/or arranging for social support to help them lose weight?	49.4%(CI95% 43.34–55.46)	<0.0001
8	What percentage of your overweight/obese patients do you assist by changing their psychiatric medications to help them lose weight?	49.2%(CI95% 43.46–54.94)	<0.0001
9	What percentage of you overweight/obese patients who are interested in losing weight do you prescribe weight loss drugs (e.g., Orlistat) to help them lose weight?	57.4%(CI95% 53.23–61.57)	<0.0001
10	What percentage of your overweight/obese patients who are interested in losing weight do you refer to outside centers (e.g., Weight Watchers, Jenny Craig, Physicians Weight Loss Centers, hospital programs, etc.)?	52.4%(CI95% 47.34–57.46)	<0.0001
11	What percentage of your overweight/obese patients who are interested in losing weight do you schedule follow-up visits to monitor for their weight control?	52.2%(CI95% 48.05–56.35)	<0.0001
12	How confident are you in your ability to do the following actions with your overweight/obese patients?	Positive *	<0.0001
13	How likely do you think it is that doing the following activities will result in your overweight/obese patients losing significant (10% or more of their initial body weight) amounts of weight?	Positive *	<0.0001
14	If you do not advise the majority of your overweight/obese patients about weight management, please identify what prevents you from doing so.	n/a **	n/a **
15	Compared to obsess patients without comorbid conditions, how often to you assist obese patients who obesity is combined with comorbid conditions such as diabetes, hypertension, and coronary artery disease to lose weight?	Positive *	<0.0001
16	The typical weight gain from psychotropic medications is…	Positive *	<0.005
17	The typical weight gain associated with the use of psychotropic medications is…	Positive *	<0.005

* Items with categorical responses; ** No statistical calculation as the item was dependent on the response to item 13, which was also a categorical response.

**Table 2:** Questionnaire Item 1 Response Frequency *.

Which of the Following Statements Best Describes Your Position toward Assisting Overweight/Obese Residents to Lose Weight? Please Check the Single Most Correct Statement.	Pretest	Posttest
Have not seriously thought about assisting my patients who are overweight/obsess to lose weight.	76%	10%
Have been thinking about assisting overweight/obese patients in losing weight within the next six months.	26%	42%
Have made formal plans to start within the next months to assist overweight/obese patients to lose weight.	0%	26%
Have been assisting overweight/obese patients to lose weight for six months or less.	0%	22%
Have been assisting overweight/obese patients to lose weight for over six months.	0%	0%
Used to assist overweight/obese patients to lose weight buy I no longer assist them with this problem.	0%	0%

* McNemar’s test (*p*-value < 0.001).

**Table 3:** Questionnaire Item 12 Response Frequency *.

How Confident Are You in Your Ability to Do the Following Actions with Your Overweight/Obese Patients?	Test	Not Confident	Slightly Confident	Moderately Confident	Confident	Highly Confident
a. **Asking** your patients if they are concerned with their weight.	Pre Post	56%	30%	8%	4%	2%
		0%	8%	16%	48%	28%
b. **Advising** your patients on weight management.	Pre Post	52%	32%	8%	4%	4%
		0%	4%	18%	58%	20%
c. **Assessing** your patients’ willingness to lose weight.	Pre Post	52%	32%	8%	8%	0%
		2%	28%	24%	38%	8%
d. **Assisting** your patients in their attempts to lose weight.	Pre Post	48%	34%	12%	6%	0%
		2%	34%	42%	22%	0%
e. **Arranging** follow-up visits for your patients.	Pre Post	44%	46%	6%	4%	0%
		6%	50%	28%	14%	2%

* McNemar’s test (*p*-value < 0.001).

Similarly, after the educational program, the participants were statistically more likely to ask their patients if they were concerned about their weight, advise patients about weight management, assess patient willingness to lose weight, and assist patients with losing weight. In terms of the ability of participants to arrange for follow-up visits for patients, most were unlikely (64%) or not sure (30%) after the educational program. Table 4 provides the item response frequency.

### 3.3. Engagement in Weight Management with Comorbid Conditions

When considering the obesity management of different patients, participants were less likely to assist obese patients with comorbid conditions, such as diabetes, before the educational program. Half the participants did not assist obese patients with losing weight before the educational program, whereas almost all participants managed patients with comorbidities as often (30%) or more often (64%) than patients without comorbidities after the educational program. Twice as many participants responded more often (34% versus 64%) after the educational program. These findings were statistically significant. Table 5 provides the item response frequency.

### 3.4. Weight Gain and Psychotropic Medication Compliance Knowledge

Participants were less familiar with patient compliance problems associated with weight gain related to psychotropic medications before the educational program. Before the program, almost all participants considered weight gain to be a minor (20%) to moderate (64%) barrier to medication compliance. After the educational program, all participants considered weight gain to be a moderate (48%) to major (52%) barrier to medication compliance; these findings were statistically significant. The recognition of this major barrier nearly tripled from 16% to 52% after the educational program. Table 6 provides the item response frequency.

**Table 5:** Questionnaire Item 15 Response Frequency *.

Compared to Obsess Patients without Comorbid Conditions, How Often to You Assist Obese Patients Who Obesity Is Combined with Comorbid Conditions Such as Diabetes?	Pretest	Posttest
Do not assist obese patients to lose weight	50%	0%
Less frequently than patients without such conditions	6%	6%
Same frequency as patients without such conditions	10%	30%
More often than patients without such conditions	34%	64%

* McNemar’s test (*p*-value < 0.0001).

**Table 6:** Questionnaire Item 17 Response Frequency *.

The Typical Weight Gain Associated with the Use of Psychotropic Medications Is:	Pretest	Posttest
A major barrier to medication compliance.	16%	52%
A moderate barrier to medication compliance.	64%	48%
A minor barrier to medication compliance.	20%	0%
Not a barrier to medication compliance.	0%	0%
Not sure.	0%	0%

* McNemar’s test (*p*-value = 0.0009).

### 3.5. Prior Education and Program Adequacy

Before the educational program, all participants reported that they had no formal education about weight management for overweight and obese patients with mental disorders (item 18). After the educational program, all participants indicated they had received formal education. In terms of the adequacy of the educational program based on the learning objectives, all participants indicated that the program was adequate or very adequate for assisting them with helping their patients with weight management (item 19).

## 4. Discussion

The purpose of this study was to increase clinicians’ awareness, change perceptions, and improve clinical practice by including weight management as part of the daily clinical care for psychiatric hospital residents. The association between mental disorders and obesity in the context of a psychiatric hospital is complicated by intensive pharmacological management, sedentary lifestyle, and poor eating habits, including the limited availability of healthy foods [19]. These obesity generating environmental factors result in psychiatric institutions being ‘obesogenic’ [32], mostly caused by the “sum of influences that the surroundings, opportunities, or conditions of life have on promoting obesity in individuals or populations” [31]. Importantly, interprofessional engagement [32] and individualized interventions [77] were reported in small qualitative studies that motivated patients in psychiatric institutions to engage in healthier lifestyles. A change in practice requires clinicians to be aware of the risks for obesity, to be able to identify obese patients, and to be willing to tailor weight management strategies with an interdisciplinary approach to person-centered care.

An important finding from this study was that all participants reported no previous education or training for the clinical management of obesity in the context of mental health. In this regard, Brown and Flint [78] noted that physicians are not properly trained to adequately address obesity, despite the availability of numerous well-established evidence-based guidelines. A national survey of non-physician health professionals (*n* = 500) in the United States reported that most nutrition professionals (78%) received high-quality weight management training during their degree program, followed by nursing (53%), exercise (50%), pharmacy (47%), and mental health (32%) professionals [79]. More than half of these professionals (*n* = 281) reported additional training in obesity management, including pharmacy (77%), nutrition (76%), nursing (59%), mental health (55%), and exercise (50%) professionals who earned continuing education credits.

Before the educational program, the clinicians did not recognize obesity as an important part of daily practice, they did not notice resident weight gain, and they lacked the prerequisite knowledge to use basic medical weight reduction strategies to manage obese residents. Similarly, mental health clinicians surveyed in different countries reported limited awareness about patient obesity and reported insufficient knowledge to manage the physical health of people with severe mental illness [80,81,82]. Most patients residing in psychiatric facilities are concerned about their weight [83] having unsuccessfully attempted to lose weight through diet and physical activity in the past and without clinician support in a highly regulated environment whose sole focus is not necessarily healthy living [38]. Continuing professional education programs in weight management should be required for clinicians practicing in psychiatric hospitals, especially if they have prescriptive authority. Furthermore, psychiatric hospital administrators should be required to participate in professional education programs focused on working with clinicians to create health living environments for patients. Clinicians, patients, and administrators each have an important role in establishing successful weight management practices in psychiatric hospitals.

Although evidence-based programs with multiple strategies [36] can address obesity across a range of mental health disorders, including bipolar disorder, depression, and schizophrenia [34,84], clinicians need help identifying at-risk residents to offer timely interventions [19]. This approach requires increased monitoring and improved medical management [28] through dietary changes accompanied by exercise and behavioral therapy. Since the participants in this study indicated that time constraints reduced their focus on resident obesity, evidence-based practices implemented by multidisciplinary teams can be used to broaden the daily focus on prevention and management [85,86,87]. Importantly, few studies were identified regarding the role of support services, such as nutritionists, clinical pharmacists, and physical therapists, for managing obesity in psychiatric hospitals [53]. In a systematic review, however, dietitian-led interventions resulted in larger effect sizes for weight loss, reduced BMI, and lower blood glucose levels [88].

The participants also encountered barriers when ordering external nutritional services and scheduling follow-up visits for specialized medical management. In this regard, psychiatric hospitals can assist clinicians by implementing clinical practice guidelines using a multidisciplinary approach to medical management. This approach would limit the environmental factors in psychiatric services [32] that can amplify the weight gain associated with medications. For example, a multidisciplinary team can limit the impact of psychiatric medications on weight gain through the incorporation of a clinical pharmacist [89] to reduce side effects and a physical therapist [90] to increase activity. If the most appropriate medication is selected during interdisciplinary rounding [91], then team members can contribute to resident lifestyle changes to reduce calorie intake, improve healthy eating, and increase physical activity [92].

After retrospective assessment of the measurement instrument and applying the new “person first language” guidelines [71,72], some items in the questionnaire were identified as potentially weight stigmatizing. For example, item #3, “What percentage of your visibly overweight/obese patients do you give a clear, strong, and personalized message urging them to lose weight?” contains stigmatizing language about weight-based stereotypes. In addition, the item requires retrospective recall that may be influenced by weight bias and may not provide an accurate estimate about the delivery of personalized obesity care. The Joint International Consensus Statement for Ending Stigma of Obesity [66] condemns “the use of stigmatizing language, images, attitudes, policies, and weight-based discrimination, wherever they occur.” As such, we believe instruments selected for measurement or assessment in obesity research should be evaluated for “person first language.”

### Limitations and Strengths

This project has five limitations and three major strengths. First, the sample size (*n* = 50) was small, but it was the target based on an *a priori* power analysis. Although response-shift bias is possible for the study design [93], observed performance-based item responses limited this concern. The potential for recall bias was also limited by spacing the pretest and posttest questionnaires by 90 days [94]. Third, the within-participant design [95] can cause fatigue effects, practice effects, carryover effects, and order effect [96]. However, this design can also increase statistical power by controlling individual differences between units within conditions, so fewer units can be used to test the same number of treatments [96,97]. Fourth, the small sample size coupled with the single study site may limit the generalizability of the findings [98]. Finally, analyzing the results of this study in comparison to other studies was limited by the scant existing literature addressing obesity management in psychiatric hospitals [98].

Despite these limitations, this study addresses an important gap in the literature specific to obesity management at psychiatric hospitals. Furthermore, the educational program was developed with expert review from the evidence-based literature, best practices, and national guidelines to help clinicians identify and effectively manage obesity. The evaluation was completed using a validated instrument. Moreover, the evaluation was conducted 90 days after the educational program to reduce recall bias and to measure planned and actual changes to clinical practice. Because the education program was tailored for a psychiatric hospital, the content may be transferable to similar institutions in other areas of the United States.

## 5. Conclusions

There is a considerable gap in the obesity literature about the medical management of obesity by clinicians in the context of psychiatric hospitals. Although evidence-based practices are reported in the literature to address gaps in obesity management in other environments, none were identified for public psychiatric hospitals. The results suggested that the educational program improved the current level of knowledge, skills, and attitudes of mental health clinicians employed at a public psychiatric hospital regarding the clinical obesity management of people living with mental health disorders. Additional research is necessary to correlate the reported behavior changes with process measures and clinical outcomes over time. This pilot study demonstrates that an inexpensive but evidence-based educational program can shift obesity management knowledge and the associated behaviors of clinicians practicing at a public psychiatric hospital.

## Figures and Tables

**Table 4:** Questionnaire Item 13 Response Frequency *.

How Likely Do You Think It Is That Doing the Following Activities Will Result in Your Overweight/Obese Patients Losing Significant Amounts of Weight?	Test	Very Unlikely	Unlikely	Not Sure	Likely	Very Likely
a. **Asking** your patients if they are concerned with their weight.	Pre Post	10%	30%	20%	34%	6%
		0%	4%	20%	42%	43%
b. **Advising** your patients on weight management.	Pre Post	16%	20%	20%	30%	14%
		0%	0%	18%	38%	44%
c. **Assessing** your patients’ willingness to lose weight.	Pre Post	20%	24%	18%	28%	10%
		0%	8%	16%	36%	40%
d. **Assisting** your patients in their attempts to lose weight.	Pre Post	28%	52%	2%	18%	0%
		0%	2%	14%	20%	64%
e. **Arranging** follow-up visits for your patients.	Pre Post	50%	34%	10%	6%	0%
		0%	64%	30%	6%	0%

* McNemar’s test (*p*-value < 0.001).

## Data Availability

The aggregated data is available from the corresponding author upon reasonable request. Individual level data may also be available upon reasonable request if the anonymity and confidentiality of the participants and the hospitals can be assured with the data release.

## Supplementary Materials

The following supporting information can be downloaded at: https://www.mdpi.com/article/10.3390/healthcare10101851/s1. There are two supplemental materials for this study. These materials include Supplemental File S1: Advising and treating overweight and obese patient questionnaire; and Supplemental File S2: Data analysis by item.
