# Supplementary material for: Improving the Physical Health of Psychiatric Hospital Residents: An Evaluation of an Obesity Education Program for Mental Health Professionals
Source: Healthcare (Basel). 2022 Sep 23;10(10):1851. doi: 10.3390/healthcare10101851 (PMC9601487; doi:10.3390/healthcare10101851)
Supplement: Supplementary file 1 [file healthcare-10-01851-s001.zip › Healthcare-1851102_Proofs_SUPP-1_2022.09.21.pdf]

Supplement File S2. Advising and Treating Overweight and Obese Patients

Directions: For each of the following statements, please circle or mark the response that most accurately describes *your opinion.* We are interested in group data only. Your individual responses will remain completely confidential. Please do not put any identifying marks on this survey.

1. Which of the following statements best describes y**our position toward assisting overweight/obese patients to lose weight**? Please check the single most correct statement.

______ I have **not seriously thought about** assisting my patients who are overweight/obsess to lose weight.

______ I **have been thinking about** assisting my overweight/obese patients in losing weight within the next six months.

______ I have **made formal plans** to start **within the next month** to assist my overweight/obese patients to lose weight.

______ I **have been assisting** my overweight/obese patients to lose weight for **six months or less**.

______ I **have been assisting** my overweight/obese patients to lose weight **for over six months**.

______ I used to assist my overweight/obese patients to lose weight, **but I no longer assist** them with this problem.

2. What percentage of your visibly overweight/obese patients do you **identify** and **document** their weight status? Please circle your response.

0% 10% 20% 30% 40% 50% 60% 70% 80% 90% 100%

3. What percentage of your visibly overweight/obese patients do you give a **clear, strong,** and **personalized message** urging them to lose weight? Please circle your response.

0% 10% 20% 30% 40% 50% 60% 70% 80% 90% 100%

4. What percentage of you overweight/obese patients do you assess whether they are **willing to make an effort** to lose weight? Please circle your response.

0% 10% 20% 30% 40% 50% 60% 70% 80% 90% 100%

5. What percentage of your overweight/obese patients, who are interested in attempting to lose weight do you or your staff use **behavioral counseling to help them lose weight**? Please circle your response.

0% 10% 20% 30% 40% 50% 60% 70% 80% 90% 100%

6. What percentage of you overweight/obese patients do you **assist by encouraging** them to use **problem-solving skills** for weight loss? Please circle your response.

0% 10% 20% 30% 40% 50% 60% 70% 80% 90% 100%

7. What percentage of your overweight/obese patients do you **assist by providing and/or arranging for social support** to help them lose weight? Please circle your response.

0% 10% 20% 30% 40% 50% 60% 70% 80% 90% 100%

Next Page

8. What percentage of your overweight/obese patients do you assist by **changing their psychiatric medications** to help them lose weight? Please circle your response.

0% 10% 20% 30% 40% 50% 60% 70% 80% 90% 100%

9. What percentage of you overweight/obese patients who are interested in losing weight do you prescribe **weight loss drugs** (e.g., Orlistat) to help them lose weight? Please circle your response.

0% 10% 20% 30% 40% 50% 60% 70% 80% 90% 100%

10. What percentage of your overweight/obese patients who are interested in losing weight do you **refer to outside centers** (e.g. Weight Watchers, Jenny Craig, Physicians Weight Loss Centers, hospital programs, etc.)? Please circle your response.

0% 10% 20% 30% 40% 50% 60% 70% 80% 90% 100%

11. What percentage of your overweight/obese patients who are interested in losing weight do you **schedule follow-up visits** to monitor for their weight control? Please circle your response.

0% 10% 20% 30% 40% 50% 60% 70% 80% 90% 100%

12. How **confident** are you in your **ability to do** the following actions with your overweight/obese patients? Please circle <u>one</u> response for each statement

**Action**	**NCA** Not confident at all	**SC** Slightly confident	**MC** Moderately confident	**C** Confident	**HC** Highly Confident
(a) **Asking** your patients if they are concerned with their weight.	NCA	SC	MC	C	HC
(b) **Advising** your patients on weight management.	NCA	SC	MC	C	HC
(c) **Assessing** your patients' willingness to lose weight.	NCA	SC	MC	C	HC
(d) **Assisting** your patients in their attempts to lose weight.	NCA	SC	MC	C	HC
(e) **Arranging** follow-up visits for your patients.	NCA	SC	MC	C	HC

13. How likely do you think it is that **doing the following** activities will result in your overweight/obese patients **losing significant** (10% or more of their initial body weight) **amounts of weight**? Please check <u>one response</u> for each statement.

(a) **Asking** your patients if they are concerned with their weight…

______ Very Unlikely ______ Unlikely ______ Somewhat Likely ______ Likely ______ Very Likely

Next Page

(b) **Advising** your patients on weight management…

______ Very Unlikely ______ Unlikely ______ Somewhat Likely ______ Likely ______ Very Likely

(c) **Assessing** your patients' willingness to lose weight…

______ Very Unlikely ______ Unlikely ______ Somewhat Likely ______ Likely ______ Very Likely

(d) **Assisting** (using weight loss medications, referring patients to outside centers) your patients in their attempts to lose weight…

______ Very Unlikely ______ Unlikely ______ Somewhat Likely ______ Likely ______ Very Likely

(e) **Arranging** follow-up visits for your residents…

______ Very Unlikely ______ Unlikely ______ Somewhat Likely ______ Likely ______ Very Likely

14. If you **do not advise** the majority of your overweight/obese patients about weight management, please identify what prevents you from doing so. Please check <u>all</u> responses that apply.

______ Fear of offending my patients.

______ It is the job of the patients' primary care physician.

______ It is the responsibility of health care professionals who specialize in nutrition.

______ Lack of clear guidelines and practice tools.

______ Constraints of time.

______ Difficulties in reimbursement.

______ Patients would be offended if I addressed reimbursement issues.

______ Limited medical training on this issue.

______ Poor patient compliance.

______ Cultural differences regarding weight and body size.

______ I do assist them in making contact with weight control centers.

______ Other (please identify ____).

15. Compared to obsess patients **without** comorbid conditions, how often to you assist obese patients who obesity is combined **with** comorbid conditions such as diabetes, hypertension, and coronary artery disease to lose weight?

______ **I do not assist** obese patients to lose weight.

______ **More often** than patients **without** such conditions.

______ The **same frequency** as patients **without** such conditions.

______ **Less frequently** than patients **without** such conditions.

Next Page

16. The typical weight gain from **psychotropic medications** is:

______ A **major** health concern.

______ A **moderate** health concern.

______ A **minor** health concern.

______ Not a health concern.

______ Not sure.

17. The typical weight gain associated with the use of **psychotropic medications** is:

______ A **major** barrier to medication compliance.

______ A **moderate** barrier to medication compliance.

______ A **minor** barrier to medication compliance.

______ Not a barrier to medication compliance.

______ Not sure.

The remaining two questions are important to understand your educational background.

18. Have you had any formal education in the weight management of clients with mental health issues?

______ Yes ______ No _____ Not Sure

If YES, where did you receive your education? (**Check all that apply**):

______ continuing education course(s)

______ university program (medical, nursing, physician assistant, etc. programs)

______ professional conferences

______ professional journals

______ other (please identify): ____.

19. How would you rate the adequacy of your professional training (clinical degree program) for how to assist your patients with weight management?

______ Not adequate at all

______ Not very adequate

______ Somewhat adequate

______ Adequate

______ Very adequate

Thank you for completing the survey. Your professional courtesy is greatly appreciated!

This survey was adapted with permission of the author – (Lichwala-Zyla et al., 2007; 2009).
