# Supplementary material for: Improving the Physical Health of Psychiatric Hospital Residents: An Evaluation of an Obesity Education Program for Mental Health Professionals
Source: Healthcare (Basel). 2022 Sep 23;10(10):1851. doi: 10.3390/healthcare10101851 (PMC9601487; doi:10.3390/healthcare10101851)

Supplement File S2. Data Analysis by Item

Bar Charts and Histograms for Participant Responses Pre- and Post-Test (n=50)

Item 1. Bar Charts for Responses

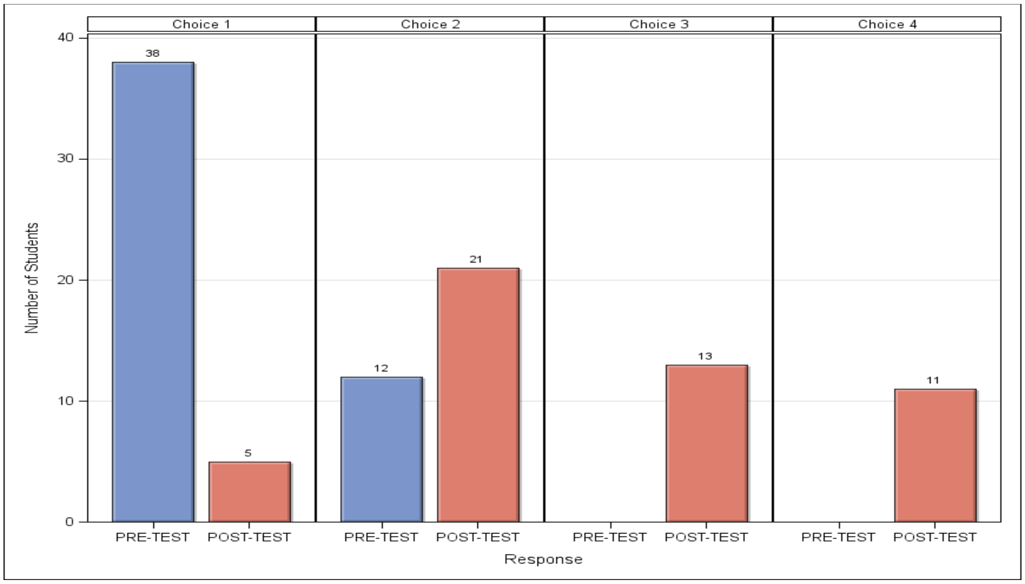

Item 2. Histogram of Responses

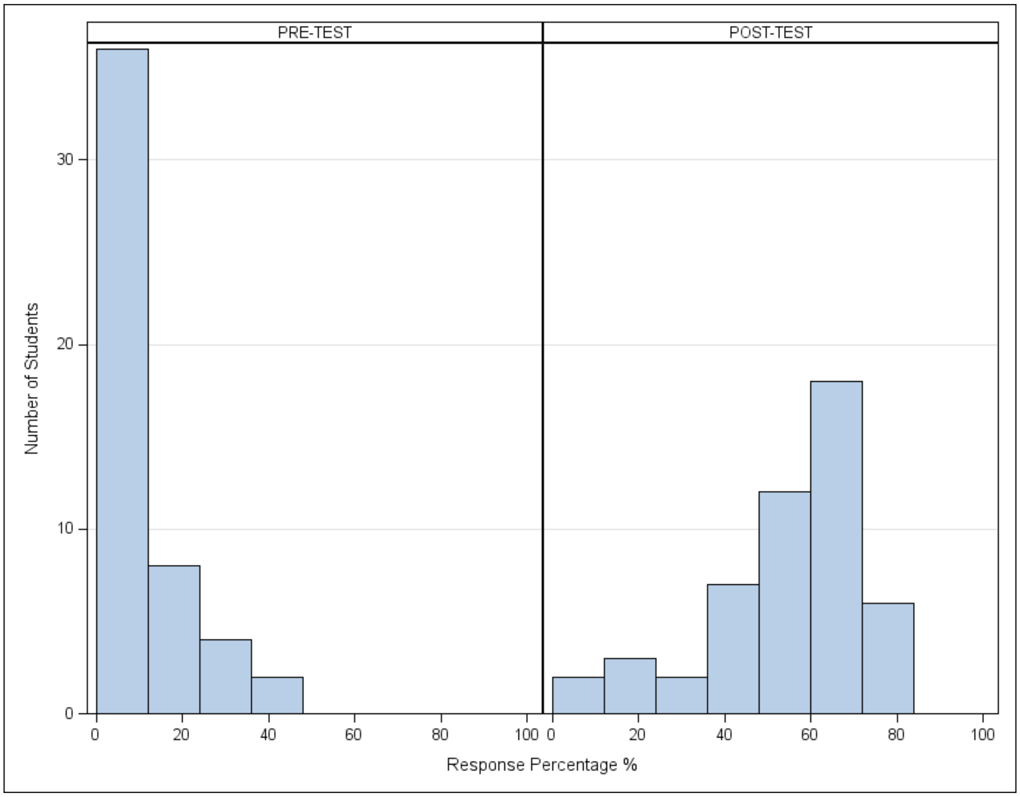

Item 3. Histogram of Responses

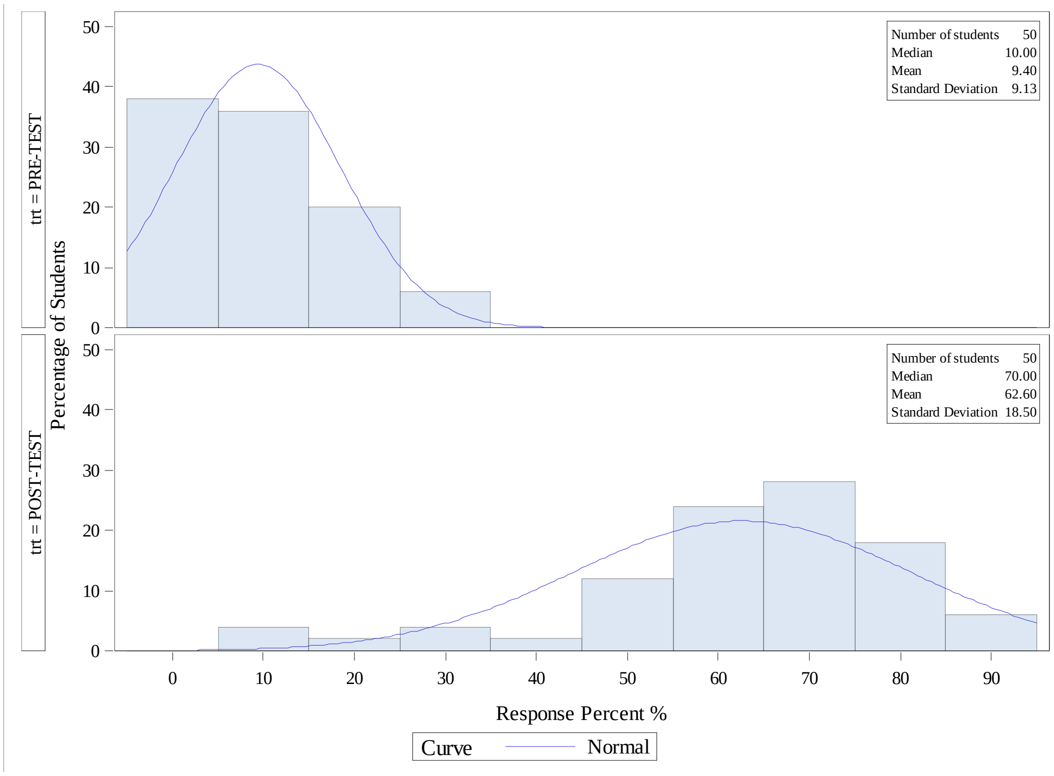

Item 4. Histogram of Responses

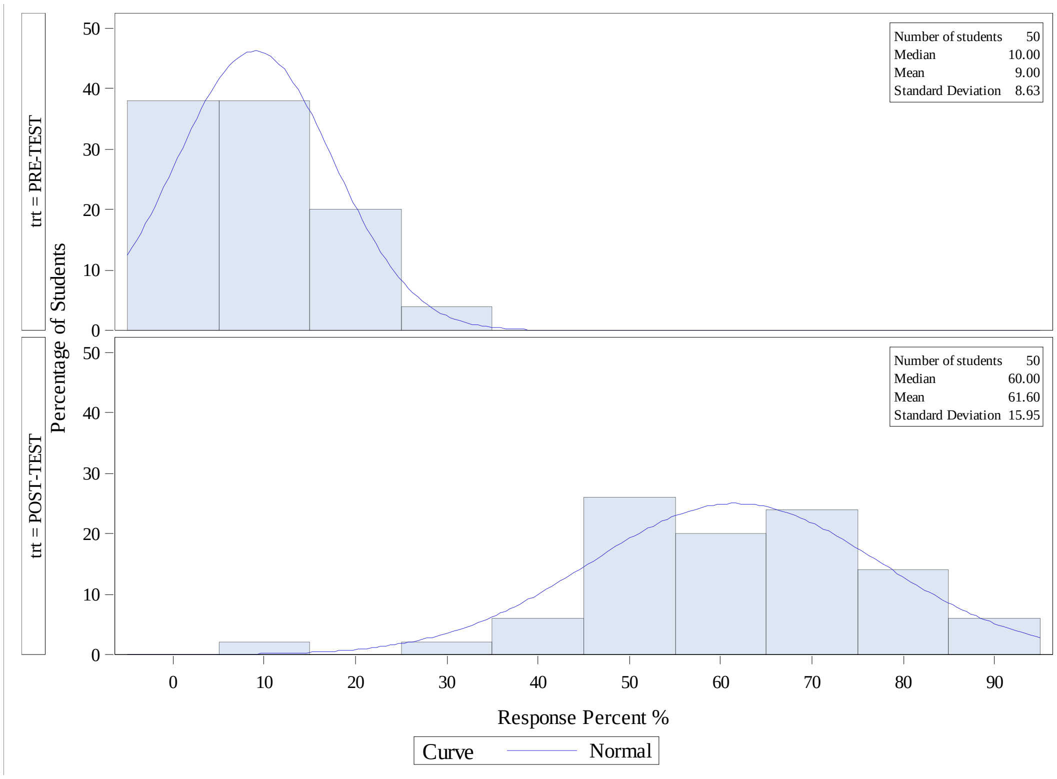

Item 5. Histogram of Responses

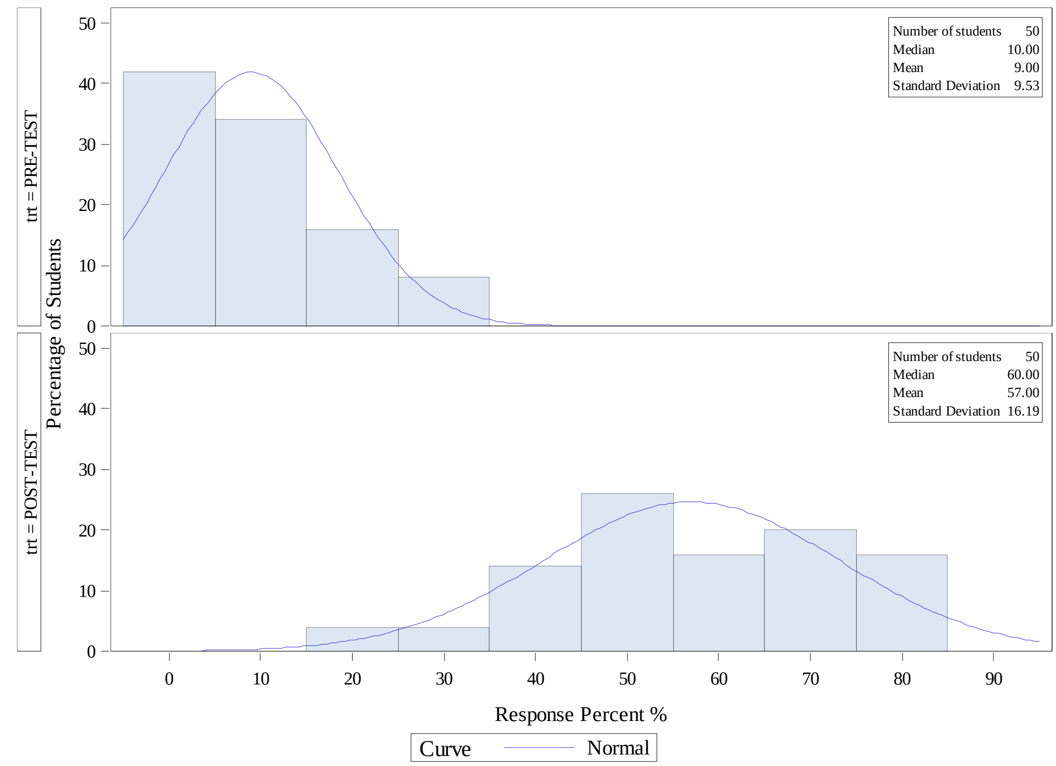

Item 6. Histogram of Responses

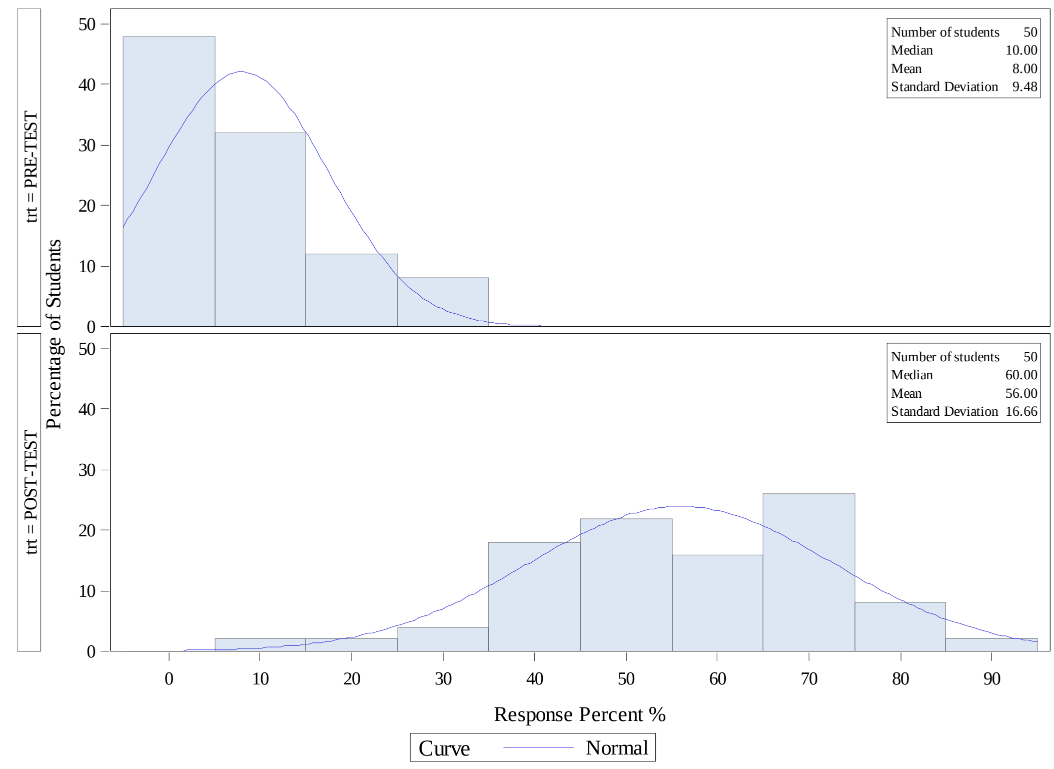

Item 7. Histogram of Responses

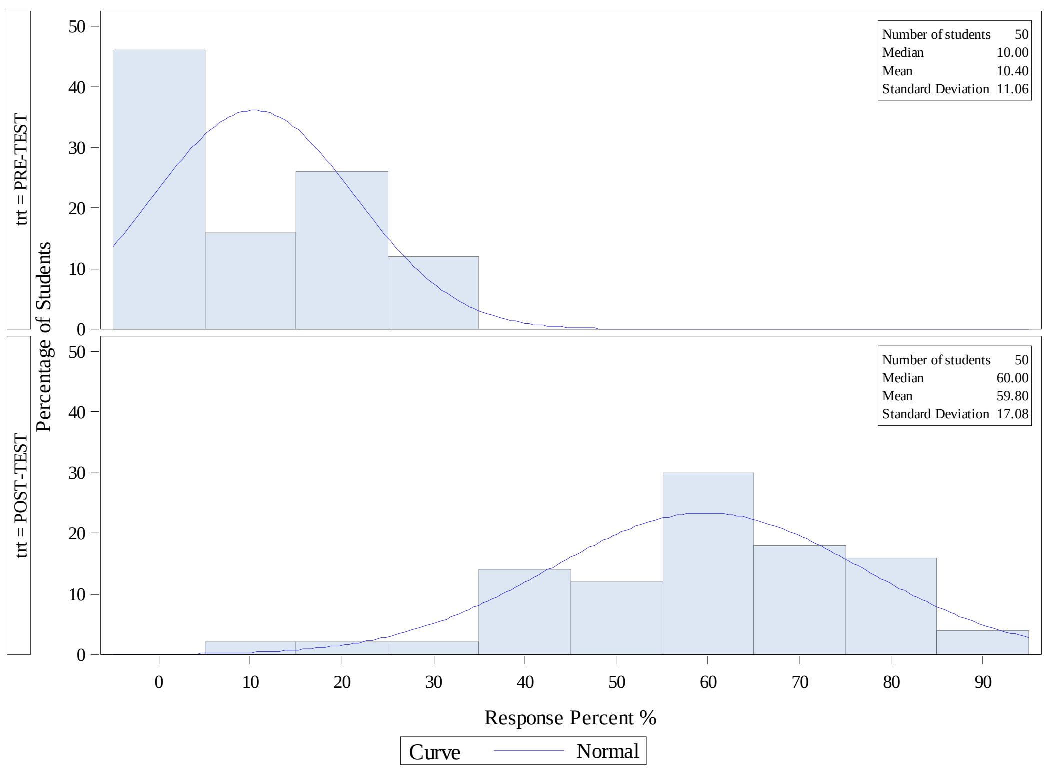

Item 8. Histogram of Responses

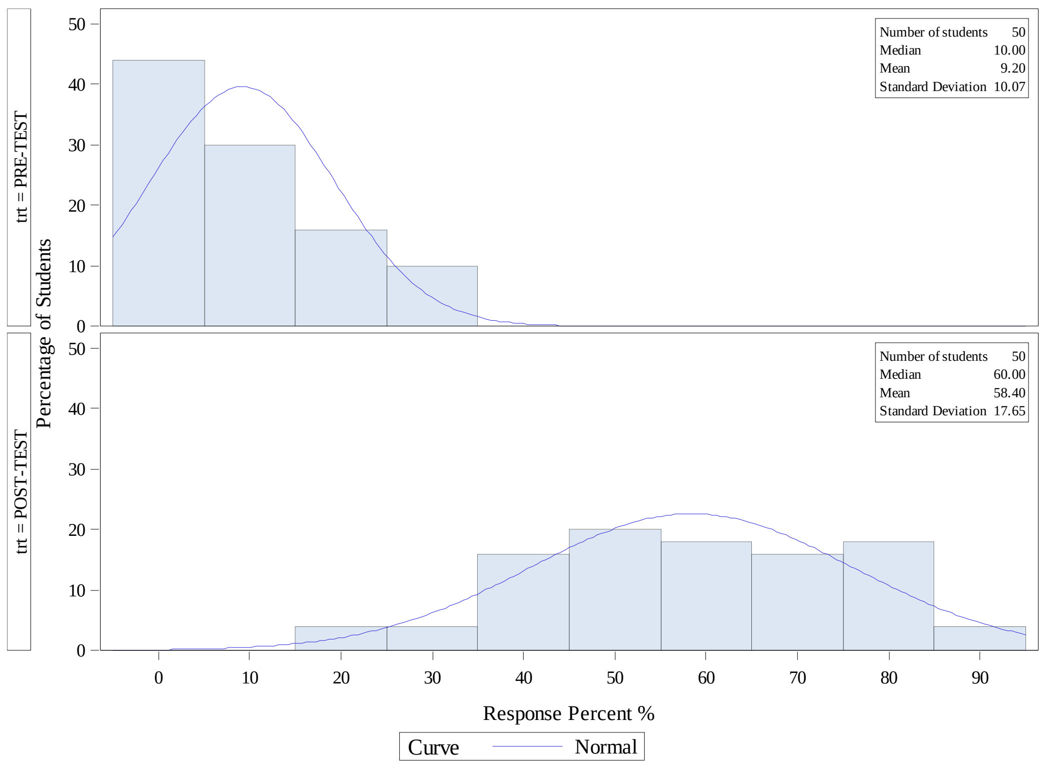

Item 9. Histogram of Responses

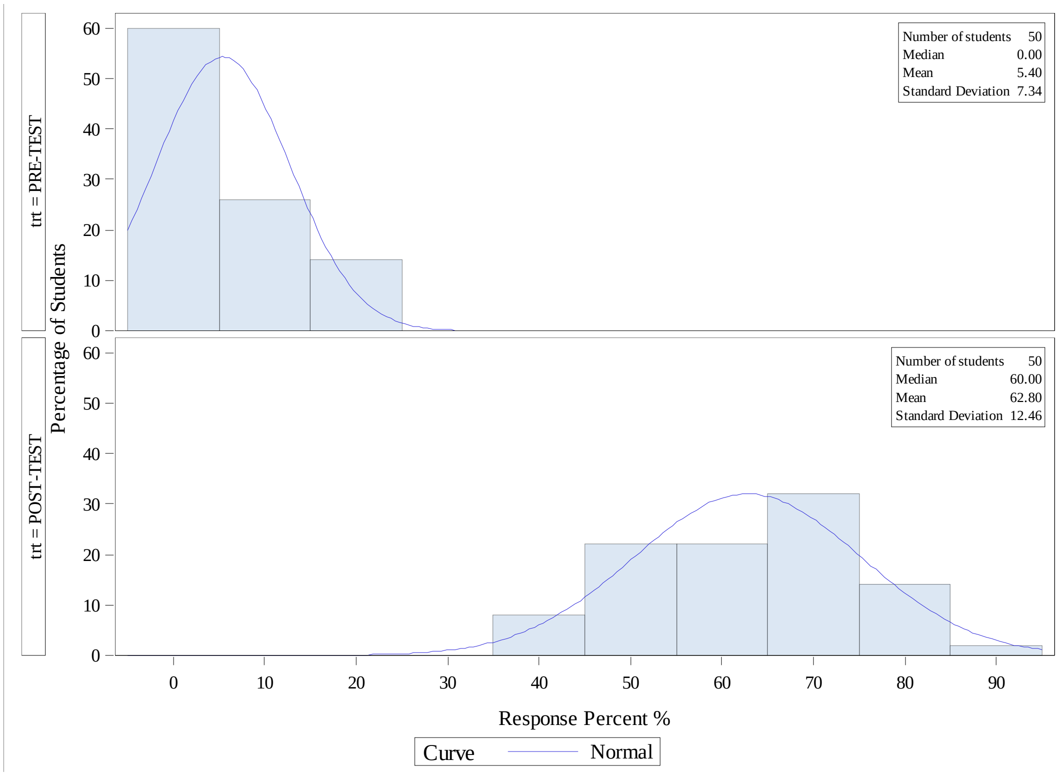

Item 10. Histogram of Responses

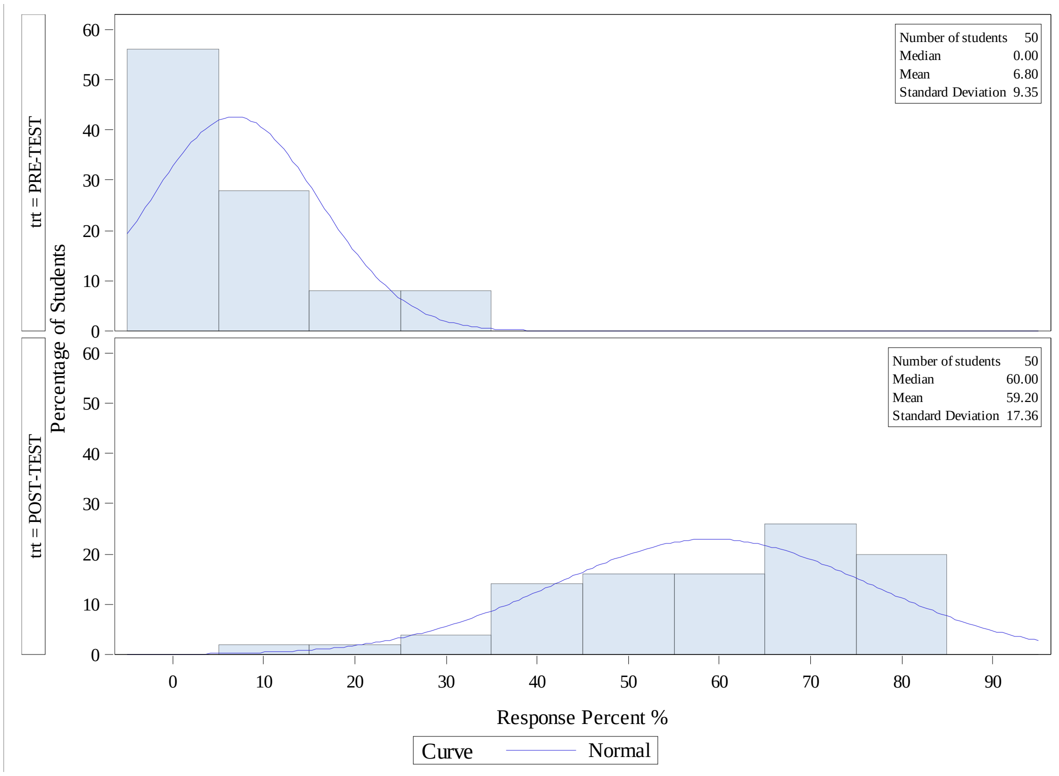

Item 11. Histogram of Responses

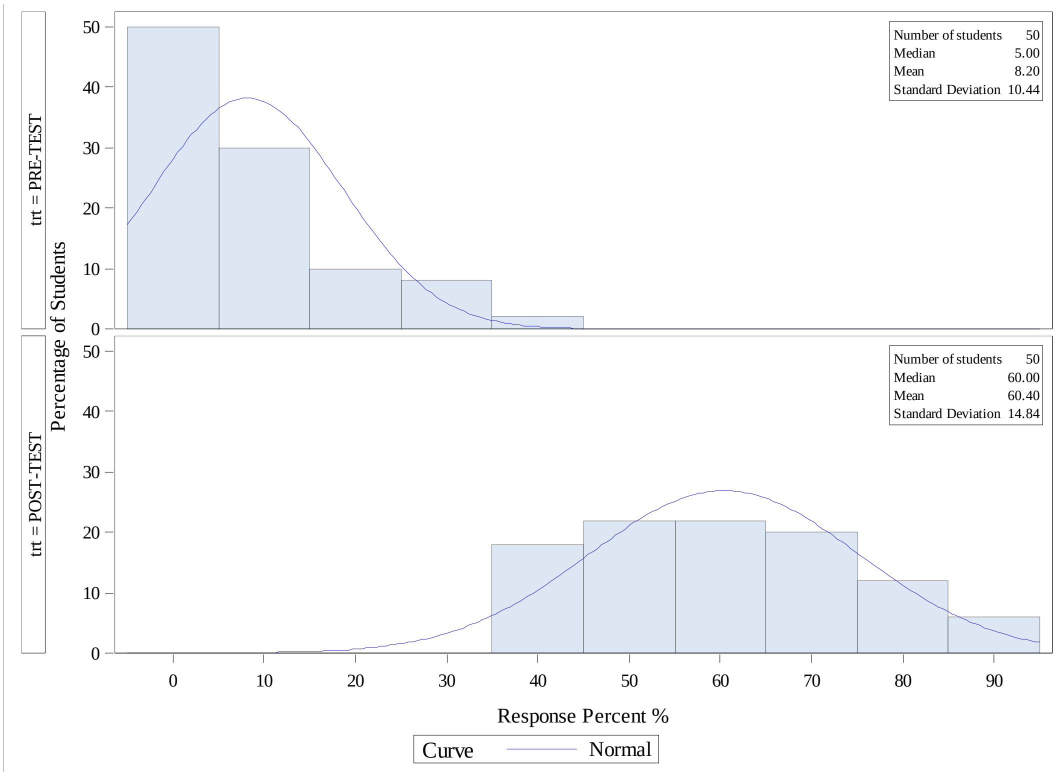

Item 12 (a, b, c, d, e). Bar Charts for Responses

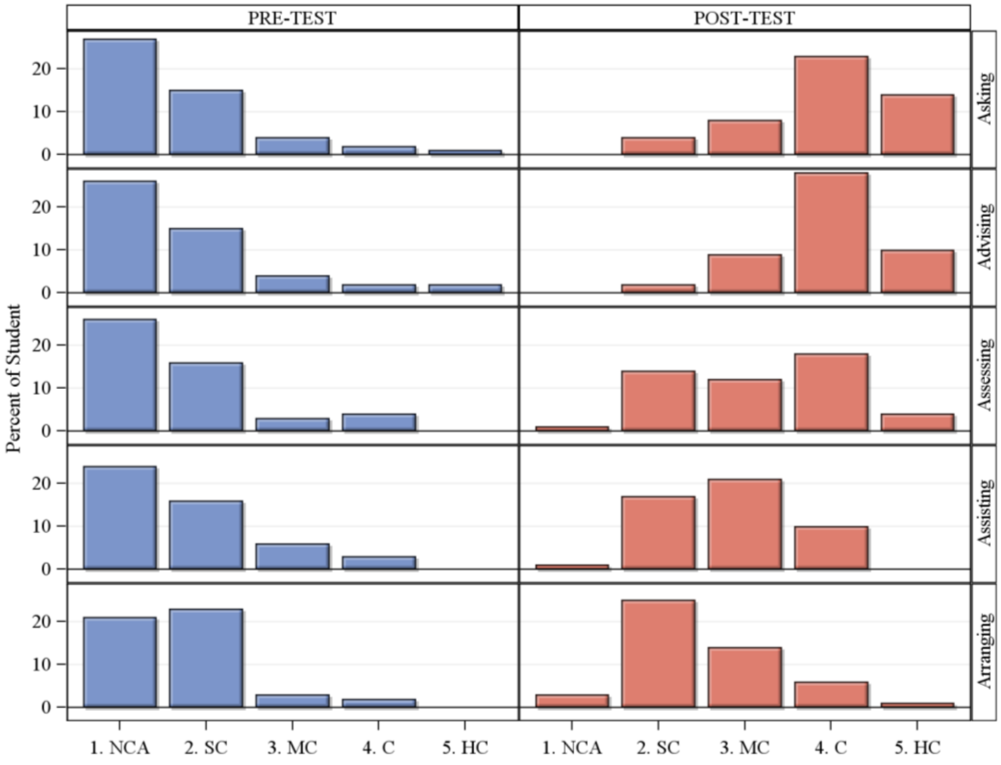

Item 13 (a, b, c, d, e). Bar Charts for Responses

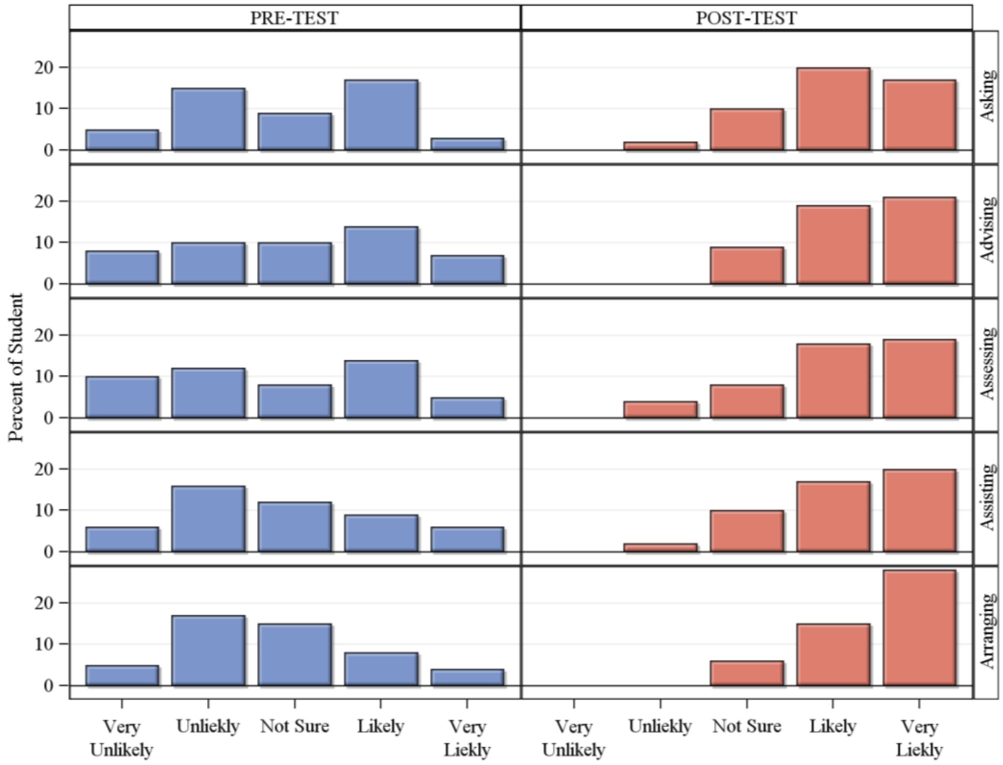

Item 15. Bar Charts for Responses

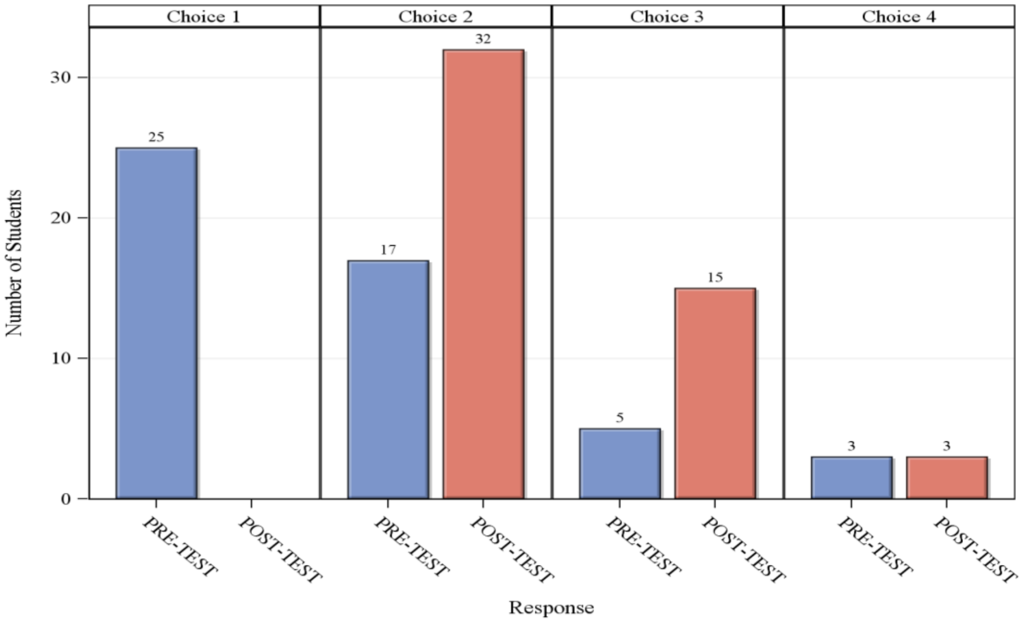

Item 16. Bar Charts for Responses

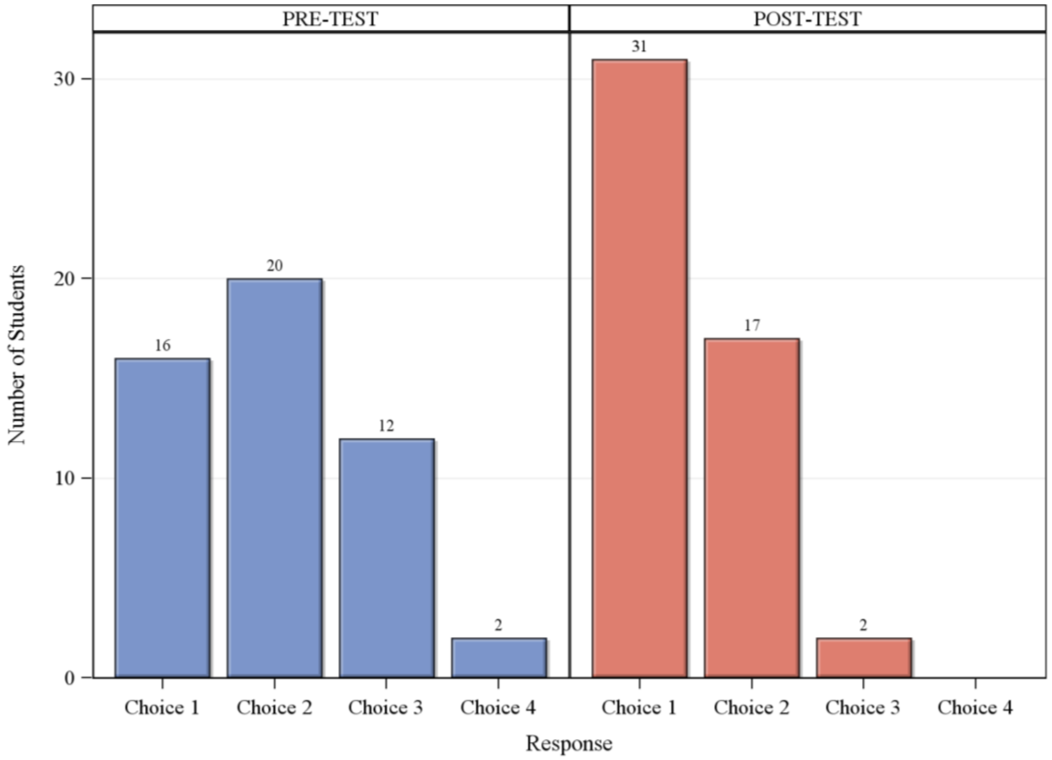

Item 17. Bar Charts for Responses

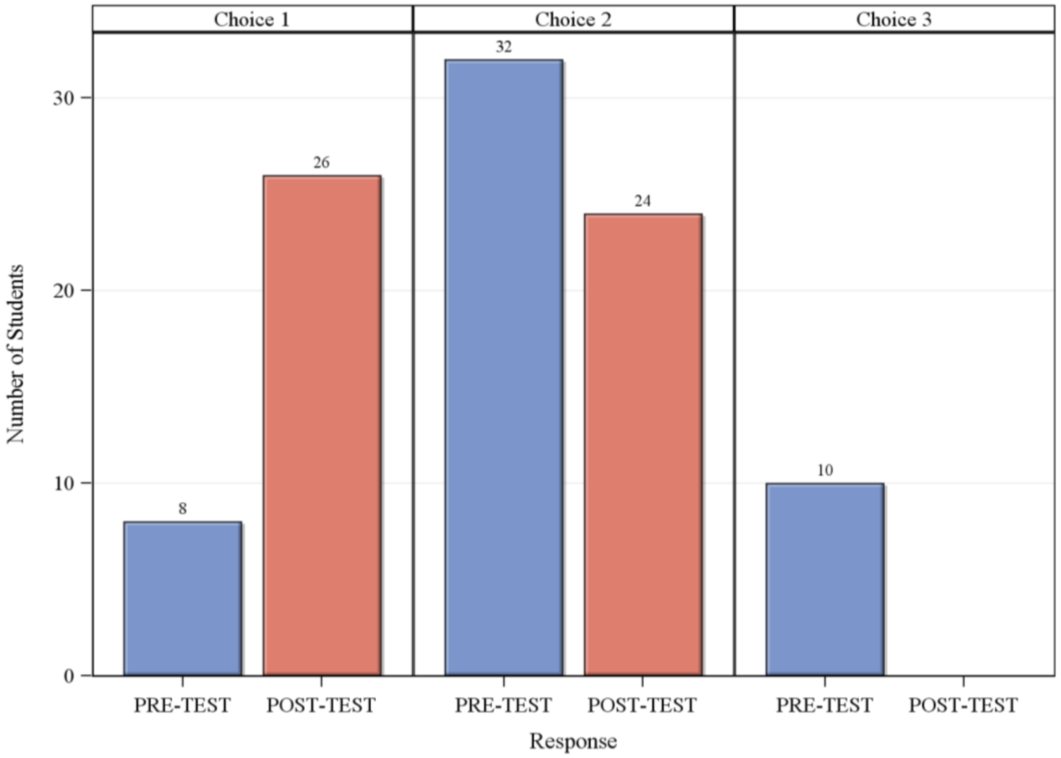

Supplement: Supplementary file 1 [file healthcare-10-01851-s001.zip › Healthcare-1851102_Proofs_SUPP-2_2022.09.21.pdf]
